# The association between neighborhood environment, prenatal exposure to alcohol and tobacco, and structural brain development

**DOI:** 10.3389/fnhum.2025.1531803

**Published:** 2025-02-18

**Authors:** Yingjing Xia, Verónica M. Vieira

**Affiliations:** Joe C. Wen School of Population and Public Health, Susan and Henry Samueli College of Health Sciences, University of California, Irvine, Irvine, CA, United States

**Keywords:** prenatal alcohol exposure, prenatal tobacco exposure, neighborhood environment, neuroimaging, child brain development

## Abstract

Prenatal alcohol and tobacco exposure affects child brain development. Less is known about how neighborhood environment (built, institutional, and social) may be associated with structural brain development and whether prenatal exposure to alcohol or tobacco may modify this relationship. The current study aimed to examine whether neighborhood environment is associated with brain volume at age 9–11, and whether prenatal exposure to alcohol or tobacco modifies this relationship. Baseline data from Adolescent Brain and Cognitive Development (ABCD) study was analyzed (*N* = 7,887). Neighborhood environment was characterized by 10 variables from the linked external dataset. Prenatal alcohol and tobacco exposures were dichotomized based on the developmental history questionnaire. Bilateral volumes of three regions of interests (hippocampal, parahippocampal, and entorhinal) were examined as outcomes. High residential area deprivation was associated with smaller right hippocampal volume. Prenatal alcohol exposure was associated with larger volume in left parahippocampal and hippocampal regions, while prenatal tobacco exposure was associated with smaller volumes in bilateral parahippocampal, right entorhinal, and right hippocampal regions. In children without prenatal tobacco exposure, high residential area deprivation was associated with smaller right hippocampal volumes. In contrast, neighborhood environment was not significantly associated with brain volumes in children with prenatal tobacco exposure. In summary, neighborhood environment plays a role in child brain development. This relationship may differ by prenatal tobacco exposure. Future studies on prenatal tobacco exposure may need to consider how postnatal neighborhood environment interacts with the teratogenic effect.

## 1 Introduction

Prenatal alcohol and tobacco exposure impacts a significant portion of children and adolescents in the US every year (Popova et al., 2017; Kondracki, 2019). It is estimated that in the United States,14.8% of pregnancies are alcohol exposed (Popova et al., 2017), and 7.1% of pregnant persons reported smoking during pregnancy (Kondracki, 2019). Children and adolescents who have been exposed to alcohol prenatally can face challenges in executive functioning, attention, language, and mental and physical health (Inkelis et al., 2020; May et al., 2021; Mattson et al., 2019). Smoking during pregnancy is associated with increased risk of stillbirth, low birth weight, small for gestational age, in addition to respiratory problems, cancers, and neurobehavioral problems in childhood (Leech et al., 1999; Gonzalez et al., 2023; Cornelius and Day, 2009).

Prenatal alcohol exposure (PAE) and prenatal tobacco exposure (PTE) are known to affect structural brain development. Both animal models and human neuroimaging studies supported that PAE is associated with smaller total brain size, and cerebrum and cerebellum volumes (Inkelis et al., 2020; Lipinski et al., 2012; Maier et al., 1997; Archibald et al., 2001; Gautam et al., 2015). Specific to cortical regions, PAE was associated with smaller white matter and grey matter volumes in the frontal, parietal, and occipital lobes (Lebel et al., 2011). Thicker cortices following PAE have been indicated in frontal, parietal and temporal regions (Donald et al., 2015; Sowell et al., 2008; Yang et al., 2012). These changes in cortical structure were associated with the functional alterations following prenatal alcohol exposure, including executive functioning and language (Gautam et al., 2015; Sowell et al., 2008). Longitudinal and cross-sectional analysis demonstrated that children with PAE have different brain-behavioral relationships over time than the children without PAE (Sowell et al., 2008; Gautam et al., 2014). Additionally, PAE was associated with alternations in the corpus callosum (Donald et al., 2015; Sowell et al., 2001; Riley et al., 1995), and subcortical structures including the basal ganglia (Mattson et al., 1996) and hippocampus (Livy et al., 2003; Gil-Mohapel et al., 2014; Sutherland et al., 1997).

Nicotine and carbon monoxide carried in tobacco smoke can disrupt fetal brain development. Nicotine can travel across the placenta and activate the nicotine acetylcholine receptors (nAChRs). Chronic exposure to exogenous nicotine can lead to desensitization of nAChRs, which is linked to poor neonatal outcomes and altered brain reward system development (Ekblad et al., 2015). Carbon monoxide in the placenta is linked to fetal hypoxia and ischemia and thereby disrupts fetal brain development (Ekblad et al., 2015). PTE was associated with smaller total brain volume, cerebral white matter volume, cerebral gray matter volume and parenchymal volume in human studies (Rivkin et al., 2008; Zou et al., 2022). Children with PTE also had smaller surface area and gyrification than children without PTE (Zou et al., 2022). In addition, PTE is linked to thinner cortices in orbitofrontal, middle frontal, and parahippocampal cortices in adolescence (Toro et al., 2008). Smaller cortical surface area in the orbitofrontal, middle frontal and anterior cingulate has been observed among children with PTE as compared to non-exposed children (Marshall et al., 2022).

Although many studies have demonstrated the effect of severe prenatal alcohol and tobacco exposure on child development, fewer studies have focused on the effect of low-to-moderate level prenatal alcohol exposure on the offsprings and the effect of prenatal alcohol exposure at low-to-moderate level are unresolved (Bandoli et al., 2023; Pyman et al., 2021). While some studies found no difference or positive relationship between low-to-moderate prenatal alcohol exposure and health (Falgreen Eriksen et al., 2012; Lundsberg et al., 2015; Chen, 2012), others found that even at low-to-moderate levels, prenatal alcohol exposure was associated with worse behavioral and brain outcomes (Long and Lebel, 2022; Williams Brown et al., 2010; Lees et al., 2020; Larkby et al., 2011). These mixed findings warrant further investigation on whether and how low-level prenatal exposure to alcohol may affect child development.

Specific to structural brain measures, a recent study showed that low-to-moderate PAE was associated with larger cerebral volumes, in contrast to earlier studies on children with severe PAE (Lees et al., 2020). It is possible that mothers who consume alcohol moderately during pregnancy have different sociodemographic characteristics and may raise their children in different neighborhood environments compared to these with more severe alcohol use. From a life-course perspective (Ben-Shlomo, 2002), exposure to physical environmental factors and social stressors throughout gestation, childhood and adolescence may cumulatively and interactively impact brain development. Therefore, it is worth examining if co-occurring environmental exposures may have contributed to the mixed findings in brain development following low-to-moderate level prenatal exposure to alcohol and/or tobacco (Lupien et al., 2018).

Neighborhood environment is a proximal and multi-dimensional construct that includes institutional infrastructure, built environment and social environment. Alcohol and tobacco use can differ by neighborhood environment due to social environment, such as exposure to violence. Self-reported perceived neighborhood safety was associated with urban women’s smoking status, and previous history of abuse or exposure to violence was a consistent factor associated with alcohol consumption during pregnancy (Patterson et al., 2012; Skagerstróm et al., 2011). Exposure to alcohol and tobacco may also differ by neighborhoods through local market availability. More stores selling alcohol and tobacco products can make them more easily accessible, and therefore increase average consumption. Indeed, systematic reviews of studies on community level alcohol availability found support for higher outlet density and higher levels of alcohol use (Bryden et al., 2012; Gruenewald et al., 2014).

Existing literature mostly focuses on examining the impact of family-level socioeconomic resources and its impact on child development. Studies supported that socioeconomic resources impact child cognitive development and academic achievement, and this association is partially mediated by alteration in structural brain development (Shaked et al., 2018; Noble et al., 2015). Lower socioeconomic resources were associated with worse performances in executive functioning, language and working memory (Evans et al., 2021; Last et al., 2018; Noble et al., 2006). Differences in brain surface area in regions supporting language, reading and executive functions mediated the pathway connecting socioeconomic status and cognitive performance (Shaked et al., 2018; Noble et al., 2015). The hypothesized mechanism is that limited socioeconomic resources in childhood increase levels of physiological stress and thus alter the development of brain structures. This cumulative stress hypothesis is supported by the elevated allostatic load of chronic stress in adolescents who experience childhood poverty (Evans et al., 2021; Brody et al., 2014).

Although family-level socioeconomic resources are clearly significant in contributing to disparities in child/adolescent brain development, only focusing on family-level factors may not be sufficient to understand the effects of environment on individual development. Historical and current inequitable policies can create systemic patterns in neighborhood environment that contribute to disparities in downstream individual-level outcomes (Scott et al., 2020; Yang et al., 2023).

Neighborhood environment may impact child brain development through social environment, institutional infrastructure, and built environment. As part of the neighborhood social environment, perceived neighborhood safety may affect child brain development due to exposure to elevated stress. Chronic exposure to glucocorticoids due to stress can interrupts the hypothalamic–pituitary–adrenal (HPA) axis functioning and thereby impacting cognition and emotional regulation (Lupien et al., 2018). Indeed, a recent study showed that adolescents whose parents had higher perceived neighborhood safety had better executive functioning (Assari et al., 2021).

Furthermore, children living in neighborhoods with more resources and better infrastructure may have better developmental outcomes. Neighborhood socioeconomic disadvantage characterizes the average socioeconomic status of a neighborhood, which is associated with physical built infrastructure and governmental services (Hirsch et al., 2017). Recent studies have connected neighborhood socioeconomic disadvantage to alterations in amygdala and the connecting white matter tract structures important for emotional processing (Bell et al., 2021; Whittle et al., 2017), as well as to acceleration of brain structure development (Rakesh et al., 2021).

Lastly, neighborhood environment may be connected to child brain development through exposure to the built environment, including air pollution, environmental noise, residential proximity to roads and traffic count. Air pollutants include ozone, carbon monoxide, sulfur dioxide, nitrogen oxide, lead, and ambient particulate matter (PM) (Costa et al., 2017). PM is characterized by aerodynamic diameter (e.g., $PM_{10}<10\;\mathrm{\mu m};PM_{2.5}<2.5\;\mathrm{\mu m},\text{Ultra}\;\text{Fine}\;\text{PM}\;\left(\text{UFPM}\right)<0.1\;\mathrm{\mu m})$ (Costa et al., 2017). PM may come from a variety of natural or anthropogenic sources. For instance, $PM_{10}$ mainly originate from road and agricultural dust, tire wear emissions, products of wood combustion, construction and demolition works, and mining operations, while $PM_{2.5}$ may be produced by oil refineries, metal processing facilities, tailpipe and brake emissions, residential fuel combustion, power plants, and wildfires (Genc et al., 2012). Air pollutant exposure has been linked to cognitive and behavioral outcomes, including worse working memory, attention, and psychomotor performances in children (Guxens et al., 2014), as well as elevated risk for Attention-Deficit and Hyperactivity Disorder (ADHD) (Aghaei et al., 2019), Alzheimer’s disease, and Parkinson’s disease (Calderón-Garcidueñas et al., 2013; Russ et al., 2019). Prenatal and/or postnatal exposure to air pollutant has been associated with alterations in corpus callosum, dentate gyrus, and cerebellum in rodent models (Rivas-Manzano and Paz, 1999; Di Domenico et al., 2020). In human neuroimaging studies, air pollutant exposure has been associated with increased activity in the frontal cortices, volumetric alterations in parietal and temporal cortices and subcortical structure such as hippocampus and putamen (Calderón-Garcidueñas et al., 2013; Binter et al., 2022; Calderón-Garcidueñas et al., 2012).

Environmental noise may affect child brain development through disturbing sleep patterns and through disrupting language learning. It is well established that even low levels of noise can produce minor fragmentation of sleep and has been associated with long-term health risks, such as cardiovascular disease (Hume et al., 2012). In adolescence, poor sleep both in quantity and quality can be associated with poorer performance in attention and executive functioning (Kuula et al., 2015). Environmental noise also can impact early language development. Noisy environment may train children to “tune out” speech, and thereby interfere with hearing and learning new words, impacting phonological aspects of language learning. A recent study found that excessive levels of noise is associated with reduced cortical thickness in the inferior frontal gyrus (Simon et al., 2022). Proximity to major roads may be associated with higher level of traffic-related air pollution, environmental noise and heat. A growing body of literature demonstrates that maternal residential proximity to major roads is associated with adverse pregnancy outcomes (Dadvand et al., 2014). Living away from major roads has been associated with greater white matter hyperintensity volume (Wilker et al., 2015).

Lastly, neighborhood walkability may be associated with child/adolescent brain development by promoting physical activity and access to neighborhood infrastructures, such as parks and libraries. A neighborhood is considered walkable if the street network is pedestrian friendly and has short transits to diverse destinations. Recent studies have found that neighborhood walkability was inversely associated with PM pollution, and was associated with white matter track diffusion properties in children (Alnæs et al., 2020).

Although the effect of severe prenatal alcohol and tobacco exposure on brain development is well-documented, no studies to date have examined the combined association between low-to-moderate prenatal exposures and neighborhood environment. The current study utilizes linked data on a US population representative cohort of child/adolescents to examine whether PAE and PTE, and neighborhood environment, are, respectively, associated with structural brain development, and whether PAE and PTE moderates the association between neighborhood environment and structural brain development, specifically the bilateral parahippocampal, hippocampal and entorhinal volumes. These regions have been previously implicated as associated with PAE and PTE. PAE has been consistently associated with emotional regulation and memory deficits, mediated by alterations in hippocampal, parahippocampal and entorhinal regions (Coles et al., 2011; Willoughby et al., 2008; Krueger et al., 2020). Similarly, PTE has been associated with worse visuospatial memory performances and alternations in related regions including the hippocampus and the parahippocampal cortex (Jacobsen et al., 2006; Puga et al., 2024; Marshall et al., 2022). Neighborhood environment factors, such as perceived neighborhood safety, may affect brain development through chronic stress exposure that disrupts the development of cognition and emotional regulation. While hippocampus is well-known for its role in episodic memory through associating various types of information, the vast connections between entorhinal cortex and the hippocampus pose the possibility that entorhinal cortex is also involved in memory formation with less specificity (Van Strien et al., 2009). The parahippocampal region plays an important role as an intermediary that reciprocally associates multimodal cortices with the hippocampus (Witter et al., 2000). Although hippocampus has long been studied for its role in episodic memory and spatial navigation, recent studies showed that hippocampus may also be crucial in emotion regulation, particularly through its connection with the amygdala (Phelps, 2004; Fallahi et al., 2024).

We hypothesize that (1) low-to-moderate PAE and PTE are associated with structural brain development; (2) neighborhood environment is associated with structural brain development; (3) low-to-moderate PAE and PTE amplifies the association between neighborhood environment and structural brain development.

## 2 Materials and methods

### 2.1 Study sample

The Adolescent Brain and Cognitive Development (ABCD) study© is a longitudinal study with 21 data acquisition sites representative of the demographic and socioeconomic composition of the United States. The data from ABCD study is public available.^1^ The goal of the ABCD longitudinal study is to determine how childhood experiences interact with each other and a child’s biology to affect brain development and other social, behavioral, academic and health outcomes.^2^

At baseline, 11,880 children between 9.0 and 10.99 years of age were recruited at random from schools in the catchment area around each of the 21 sites. Data collected include developmental history questionnaire and magnetic resonance imaging. The study will follow participants for 10 years, with annual and biannual follow-up visits according to the assessment of interests (Garavan et al., 2018).

The current analysis utilized baseline data. Among the 11,880 children initially enrolled in the study, 12 later withdrew their consent. We excluded twins and triplets (*n* = 2,179) and then randomly selected one sibling from families that have multiple children enrolled in the study (*n* = 926) to avoid dependencies between participants. We excluded site 22 for small sample size (*n* = 32). Lastly, we also excluded neuroimaging scans that did not pass neuroimaging quality checking for motion, image artifacts, or inaccuracy in surface reconstruction (*n* = 844). The final sample size is 7,887 participants.

### 2.2 Measures

#### 2.2.1 Neuroimaging measures

The ABCD study employs an imaging protocol that is harmonized across Siemens, GE, and Philips 3 T platforms. The hardware, imaging sequence and data acquisition parameters have been described in detail elsewhere (Casey et al., 2018; Hagler et al., 2019). The current analysis focused on cortical volumes extracted from the T_1w_ acquisition. Briefly, the T_1w_ acquisition was a 3D T_1w_ inversion prepared RF-spoiled gradient echo scan with prospective motion correction when available. Motion correction and other data processing procedures have been described elsewhere (Hagler et al., 2019). Cortical volumes of 34 bilateral regions in the Desikan-Killiany atlas were extracted using the FreeSurfer v5.3. We only included scans that satisfied quality control criteria recommended by ABCD’s Data Analytics, Informatic and Resource Center (DAIRC) for T_1w_ and T_2w_ scans. The quality control criteria include manual review for artifacts, data quality and incidental findings (Hagler et al., 2019).

The current analysis focused on bilateral volumes of three regions: entorhinal cortex, parahippocampal cortex, and hippocampus. These three regions of interest were selected due to their demonstrated significance to memory and emotional regulation. The volumes of each region were examined by hemisphere (left vs. right).

#### 2.2.2 Neighborhood environment

Neighborhood environment measures were acquired from the linked external dataset in ABCD data release 5.0. ABCD researchers established a curated Geographic Information System (GIS) database *a priori* with the goal to minimize exposure misclassification by matching spatial and temporal domains. Participants’ primary, secondary, and tertiary residential addresses were collected at baseline from the caregivers between October 2016 to October 2018. ABCD DAIRC geocoded the longitude and latitude of baseline addresses using Google Maps Application Programming Interface. Values from the GIS database were assigned to each participants based on the longitude and latitude of their baseline addresses (for more details, see Fan et al., 2021).

Neighborhood environment was characterized by 10 variables: proximity to roads (U.S. Geological Survey, 2022), walkability index (U.S. Environmental Protection Agency, 2024), traffic count (U.S. Geological Survey, 2022), NO_2_ (di et al., 2019), Ozone (Requia et al., 2020), PM_2.5_ (di et al., 2019), lead-exposure risk (Washington State Department of Health, 2018), Area Deprivation Index (ADI) (Kind et al., 2014), parent-report neighborhood safety (Echeverria, 2004) and environmental noise (Mennitt et al., 2013; Mennitt and Fristrup, 2016) (Table 1). We selected these 10 variables to characterize both the neighborhood social environment and built environment. At the time of this analysis, only NO_2_, PM_2.5_, and Ozone data were available for the birth address. Correlations between birth address linked data and baseline address linked data are large for NO_2_ [*r*(4954) = 0.576] and PM_2.5_ [*r*(4954) = 0.551], but small for Ozone [*r*(4954) = −0.035]. Ozone concentration can vary greatly by time at the same location due to meteorological conditions (Lu et al., 2019). Due to limited availability of data linked spatially to birth addresses, we chose to use linked data from baseline addresses in our analysis.

**Table 1:** Characteristics of neighborhood environment variables.

Definition	Description	Data source/citation	Temporal resolution	Spatial resolution
Proximity to major roads	Number of meters away from major road or highway.	https://nationalmap.gov/small_scale/mld/1roadsl.html	Estimate from 2016	Address point
National Walkability index	Composite index ranking census block groups according to their walkability.	https://www.epa.gov/smartgrowth/smart-location-mapping#walkability	Estimate from 2010	Census tract
Traffic count	Traffic counts modeled at the 1 km^2^ resolution.	https://nationalmap.gov/small_scale/mld/1roadsl.html	Estimate from 2016	Address point
NO_2_	Spatio-temporal model predictions measured in ppb (parts per billion) 1 km^2^ resolution.	Di Domenico et al. (2020)	Annual average of daily estimates, maximum and minimum daily level in 2016	Address point
PM_2.5_	Spatio-temporal model predictions measured in μg/m^3^ at 1 km^2^ resolution.	Di Domenico et al. (2020)	Annual average of daily estimates, maximum and minimum daily level in 2016	Address point
O3	Spatio-temporal model predictions measured in ppb (parts per billion) 1 km^2^ resolution.	Requia et al. (2020)	Annual average of daily estimates, maximum and minimum daily level in 2016	Address point
Lead exposure	Imputed estimate of lead exposure based on age of homes and poverty levels in census tract.	Washington Tracking Network, Washington State Department of Health. Childhood lead risk map. https://fortress.wa.gov/doh/wtn/WTNPortal/	Average of annual estimates spanning 2010–2014	Census tract
Area deprivation index (ADI)	Composite index of a census tract’s socioeconomic disadvantage based on income, education, employment, and housing quality using data from the American Community Survey. National percentile.	Kind et al. (2014)	Average of annual estimates spanning 2010–2014	Census tract
Parent-report neighborhood safety	Sum of three Likert scale questions on perceived neighborhood safety self-reported by the caregivers. Questions include I feel safe walking in my neighborhood, day or night. Violence is not a problem in my neighborhood. My neighborhood is safe from crime.	Echeverria (2004)	Collected at Baseline	
Environmental Noise	The day-night average sound level over 24-h period where sound from 10 pm – 7 pm is upweighted by 10 dB	Mennitt et al. (2013) and Mennitt and Fristrup (2016)	Modeled based on acoustic data during 2000–2004	270 m resolution

ADI, area deprivation index; ppb, parts per billion; PM, particulate matter; NO_2_, nitrogen dioxide; O_3_, ozone.

#### 2.2.3 Prenatal alcohol and tobacco exposure

Prenatal alcohol and tobacco exposure data were collected retrospectively from caregivers at baseline. Caregivers were asked if there was alcohol/tobacco use before and after pregnancy recognition. If a caregiver answered “yes” to alcohol use (before or after pregnancy recognition), the caregiver was then asked about the estimated average number of drinks per week and the maximum number of drinks consumed per occasion. If a caregiver answered “yes” to tobacco use (before or after pregnancy recognition), the caregiver was then asked about the number of cigarettes smoked per day. On average, the caregivers reported consuming 3.9 drinks per week (SD = 4.3) before pregnancy recognition and the consumption dropped to 1.9 drinks per week (SD = 4.3) after pregnancy recognition. The maximum number of drinks consumed per occasion also dropped from 2.4 drinks per occasion (SD = 1.5) before pregnancy recognition to 1.3 drinks per occasion (SD = 1.4) after pregnancy recognition. The average number of cigarettes per day only dropped slightly from before to after pregnancy recognition, from 8.2 cigarettes per day (SD = 6.3) to 7.4 cigarettes per day (SD = 5.8).

Both prenatal alcohol and tobacco exposures were characterized as binary variables, where a participant was considered exposed if their caregiver endorsed maternal use of alcohol/tobacco during pregnancy either before or after pregnancy recognition. A participant was only considered to be not exposed if the caregiver answered no to maternal alcohol/tobacco use during pregnancy for both before and after pregnancy recognition.

#### 2.2.4 Individual-level covariates

The covariates adjusted in the analysis were chosen based on the construction of a Directed Acyclic Graph (DAG). We adjusted for age in months, sex assigned at birth, child race (Asian American/Pacific Islander, Alaskan Native/American Indian, Black/African American, White, Other), ethnicity (Hispanic, non-Hispanic), whether the family is below federal poverty line (Yes/No), family education (less than high school, high school/GED, some college/Bachelor’s, graduate degrees), and exposure to any substances prenatally other than alcohol or tobacco (Yes/No). The use of other substances during pregnancy was also reported, including marijuana, cocaine/crack, heroine/morphine, and oxycontin. The number and proportion of participants with prenatal exposure to each of these substances are reported in the Supplementary Tables 1, 2. We combined all other prenatal substance use (other than alcohol and tobacco) into one dichotomous variable. We calculated whether a family was below federal poverty line using a combination of family income and household size (Rakesh et al., 2021).

### 2.3 Statistical analysis

To examine whether prenatal alcohol exposure and prenatal tobacco exposure were associated with structural brain development, we stratified by hemisphere (left and right). Within each stratum, we used linear mixed effect models with the brain volumes as outcomes, PAE, PTE and individual-level covariates as fixed effects, and a random intercept for each study site. We used Benjamini-Hochberg false discovery rate (FDR) correction to adjust for multiple comparison across regions of interests.

Similarly, to examine whether neighborhood environment was associated with structural brain development, we used linear mixed effect models with the brain volumes as outcomes, neighborhood environment variables and individual-level covariates as fixed effects, and a random intercept for each study site stratified by hemispheres. Because the neighborhood environment variables were on disparate scales, the variables were centered and standardized to have mean 0 and variance 1 to facilitate model convergence. FDR correction was used to account for multiple comparison across regions of interests.

Lastly, to examine whether neighborhood environment interacted with PAE or PTE, we stratified by prenatal exposure. We chose stratification for a more straightforward interpretation of the models. Within each stratum (prenatal exposure x hemisphere), we used linear mixed effect models with the brain volumes as outcomes, neighborhood environment variables and individual-level covariates as fixed effects, and a random intercept for each site. In analyses where the data did not support random effects, we removed the random intercepts.

All analyses were conducted using R (R development Core Team, 2019; Loy and Hofmann, 2014; Kuznetsova et al., 2017; Heinzen et al., 2021; Friedman et al., 2010; Bates et al., 2015; Wickham et al., 2022; Wickham et al., 2019; Wickham, 2016). This study was exempt from Institutional Review Board review.

## 3 Results

### 3.1 Demographics

The mean age of the study sample was 118.7 months, or 9.8 years (SD = 7.5 months). Forty-eight percent of the total sample were assigned female at birth. The sample was on average highly educated. The mean family education in years was 17 (SD = 2.5 years). Eighteen percent of the sample participants lived below federal poverty line. Forty percent of the study sample had a family income of 100,000 US dollars and above.

On average, the sample did not differ by prenatal alcohol exposure in age and sex (Table 2). Participants who were exposed to alcohol prenatally were more likely to White, not identify as Hispanic, having slightly higher family education [PAE: Mean (SD) = 18.0 (1.9); no PAE: Mean (SD) = 17.2 (2.7)], higher family income, more likely to have prenatal exposure to other substances besides alcohol or tobacco [PAE: *n* = 211 (3.9%); no PAE: *n* = 345 (16.9%)] and less likely to be living below federal poverty line than their peers who were not exposed to alcohol prenatally.

**Table 2:** Demographic characteristics of the study sample by prenatal alcohol exposure.

	No exposure (*N* = 5,384)	Exposed (*N* = 2041)	Total (*N* = 7,425)	*p*-value
Age (months)				0.505
Mean (SD)	118.8 (7.4)	118.6 (7.5)	118.7 (7.5)	
Biological sex assigned at birth (Female)	2,545 (47.3%)	1,001 (49.0%)	3,546 (47.8%)	0.174
Missing	1 (0.0%)	0 (0%)	1 (0.0%)	
Ethnicity (Hispanic)	1,366 (25.7%)	341 (16.9%)	1707 (23.3%)	< 0.001
Missing	71 (1.3%)	23 (1.1%)	94 (1.3%)	
Race				< 0.001
Asian American and Pacific Islander	384 (7.3%)	116 (5.7%)	500 (6.8%)	
Alaska Native/American Indian	148 (2.8%)	52 (2.6%)	200 (2.7%)	
Black/African American	1,100 (20.8%)	267 (13.1%)	1,367 (18.7%)	
Other Race	446 (8.4%)	121 (6.0%)	567 (7.8%)	
White	3,201 (60.6%)	1,477 (72.7%)	4,678 (64.0%)	
Missing	105 (2.0%)	8 (0.3%)	113 (1.5%)	
Family education (years)				< 0.001
Mean (SD)	17.2 (2.7)	18.0 (1.9)	17.4 (2.5)	
Missing	1,188 (22.1%)	375 (18.4%)	1,563 (21.1%)	
Below federal poverty line (Yes)	1,004 (21.4%)	197 (10.3%)	1,201 (18.2%)	< 0.001
Missing	690 (12.8%)	127 (6.2%)	817 (11.0%)	
Use any other drug besides alcohol or tobacco during pregnancy (Yes)	211 (3.9%)	345 (16.9%)	556 (7.5%)	< 0.001
Missing	47 (0.9%)	53 (2.6%)	100 (1.3%)	
Family income				< 0.001
<$5,000	246 (5.1%)	32 (1.7%)	278 (4.1%)	
$5,000–$11,999	232 (4.8%)	43 (2.2%)	275 (4.1%)	
$12,000–$15,999	160 (3.3%)	35 (1.8%)	195 (2.9%)	
$16,000–$24,999	279 (5.8%)	66 (3.4%)	345 (5.1%)	
$25,000–$34,999	367 (7.6%)	88 (4.5%)	455 (6.7%)	
$35,000–$49,999	480 (9.9%)	112 (5.8%)	592 (8.7%)	
$50,000–$74,999	658 (13.6%)	250 (12.9%)	908 (13.4%)	
$75,000–$99,999	681 (14.1%)	301 (15.6%)	982 (14.5%)	
$100,000–$199,999	1,289 (26.7%)	694 (35.9%)	1983 (29.3%)	
$200,000 and greater	439 (9.1%)	314 (16.2%)	753 (11.1%)	
Missing	553 (10.3%)	106 (5.2%)	659 (8.8%)	

Four hundred and sixty-two of the 7,887 participants were missing prenatal alcohol exposure data.

The study sample did not differ by prenatal tobacco exposure on average in age and sex (Table 3). Participants who were exposed to tobacco prenatally were less likely to identify as Hispanic or White, having a slightly lower family education [PTE: Mean (SD) = 16.2 (2.2) years; no PTE: Mean (SD) = 17.6 (2.5) years], more likely to have prenatal exposure to other substances besides alcohol or tobacco [PTE: *n* = 223 (3.4%); no PTE: *n* = 332 (31.2%)] and more likely to live below federal poverty line.

**Table 3:** Demographic characteristics of the study sample by prenatal tobacco exposure.

	No exposure (*N* = 6,612)	Exposed (*N* = 1,063)	Total (*N* = 7,675)	*p*-value
Age (months)				0.116
Mean (SD)	118.7 (7.4)	119.0 (7.6)	118.7 (7.4)	
Biological sex assigned at birth (Female)	3,146 (47.6%)	517 (48.6%)	3,663 (47.7%)	0.525
Missing	1 (0.0%)	0 (0.0%)	1 (0.0%)	
Ethnicity (Hispanic)	1,553 (23.8%)	195 (18.7%)	1748 (23.1%)	< 0.001
Missing	78 (1.2%)	18 (1.7%)	96 (1.3%)	
Race				< 0.001
Asian American and Pacific Islander	475 (7.3%)	43 (4.1%)	518 (6.9%)	
Alaska Native/American Indian	151 (2.3%)	56 (5.3%)	207 (2.7%)	
Black/African American	1,084 (16.7%)	309 (29.3%)	1,393 (18.4%)	
Other Race	508 (7.8%)	67 (6.4%)	575 (7.6%)	
White	4,289 (65.9%)	578 (54.9%)	4,867 (64.4%)	
Missing	105 (1.6%)	10 (0.1%)	115 (1.5%)	
Family education (years)				< 0.001
Mean (SD)	17.6 (2.5)	16.2 (2.2)	17.5 (2.5)	
Missing	1,259 (19.0%)	344 (31.4%)	1,603 (20.1%)	
Below federal poverty line (Yes)	922 (15.5%)	295 (32.6%)	1,217 (17.8%)	< 0.001
Missing	675 (10.2%)	157 (14.8%)	832 (10.8%)	
Use any other drug besides alcohol or tobacco during pregnancy (Yes)	223 (3.4%)	332 (31.2%)	555 (7.2%)	< 0.001
Missing	80 (1.2%)	50 (4.7%)	130 (1.7%)	
Family Income				< 0.001
<$5,000	208 (3.4%)	72 (7.7%)	280 (4.0%)	
$5,000–$11,999	193 (3.2%)	85 (9.1%)	278 (4.0%)	
$12,000–$15,999	150 (2.5%)	46 (4.9%)	196 (2.8%)	
$16,000–$24,999	282 (4.6%)	68 (7.3%)	350 (5.0%)	
$25,000–$34,999	358 (5.9%)	108 (11.5%)	466 (6.7%)	
$35,000–$49,999	493 (8.1%)	127 (13.6%)	620 (8.9%)	
$50,000–$74,999	799 (13.2%)	147 (15.7%)	946 (13.5%)	
$75,000–$99,999	904 (14.9%)	112 (12.0%)	1,016 (14.5%)	
$100,000–$199,999	1931 (31.8%)	142 (15.2%)	2073 (29.6%)	
$200,000 and greater	747 (12.3%)	29 (3.1%)	776 (11.1%)	
Missing	547 (8.3%)	127 (11.9%)	674 (8.8%)	

Two hundred and twelve of the 7,887 participants were missing prenatal tobacco exposure data.

### 3.2 Associations between neighborhood environmental exposure and brain volumes

For the left hemisphere (Table 4), ADI was statistically significant for entorhinal volume (b[95% CI] = −18.087[−33.577, −2.552], *p* < 0.05) and hippocampal volume (b[95% CI] = −22.912[−38.423, −6.446], *p* < 0.01), but neither remained significant after FDR correction. Higher area deprivation was associated with smaller volumes in the entorhinal and hippocampal regions.

**Table 4:** Estimates and 95% confidence intervals of neighborhood environment exposure baseline, adjusting for age in months, sex assigned at birth, child race, ethnicity, whether the family is below federal poverty line, family education, and exposure to any substances prenatally other than alcohol or tobacco.

	Left hemisphere volumes			Right hemisphere volumes		
	Para-hippocampal	Entorhinal	Hippocampal	Parahippocampal	Entorhinal	Hippocampal
Proximity to major roads	−6.63 [−16.72, 4.02]	−0.42 [−11.88, 11.62]	2.38 [−8.57, 13.26]	−6.69 [−15.42, 2.08]	−0.12 [−11.64, 11.59]	3.34 [−8.15, 14.79]
National walkability index	1.46 [−12.75, 14.29]	4.21 [−10.77, 19.14]	6.32 [−8.07, 20.93]	7.51 [−4.13, 19.06]	4.03 [−11.09, 19.57]	8.70 [−6.45, 24.26]
Traffic count	−4.56 [−16.02, 6.54]	3.08 [−10.52, 15.44]	1.71 [−10.34, 13.69]	−2.10 [−11.72, 7.53]	7.81 [−5.13, 20.42]	2.93 [−9.69, 15.60]
NO_2_	−7.66 [−21.82, 5.33]	−2.80 [−17.58, 11.95]	6.27 [−9.77, 22.11]	−1.26 [−14.09, 11.29]	9.59 [−6.74, 25.89]	9.98 [−7.33, 27.08]
PM_2.5_	3.66 [−10.14, 17.54]	2.10 [−13.31, 18.05]	10.72 [−6.01, 26.98]	−5.38 [−18.43, 7.61]	−5.92 [−22.86, 10.93]	7.014 [−10.82, 24.51]
O_3_	4.19 [−7.93, 14.72]	−9.50 [−23.15, 2.41]	6.40 [−5.59, 18.57]	4.74 [−4.80, 14.56]	−3.55 [−16.20, 9.12]	1.01 [−11.70, 13.97]
Lead exposure	2.11 [−9.79, 15.37]	8.11 [−5.79, 21.98]	6.05 [−7.32, 19.52]	−0.29 [−10.88, 10.74]	12.42 [−1.85, 26.48]	10.26 [−4, 24.37]
Area deprivation index	−7.28 [−20.76, 6.89]	−18.09 [−33.58, −2.55] *	−22.91 [−38.42, −6.45] **	−10.35 [−22.73, 2.81]	−21.44 [−37.61, −4.72]*	−32.40 [−48.96, −14.95]^***^, ^†^
Parent-report neighborhood safety	−7.93 [−19.61, 4.45]	3.79 [−9.65, 17.53]	−3.71 [−16.23, 9.00]	−7.08 [−17.09, 3.18]	−2.05 [−15.39, 11.46]	−3.70 [−16.86, 9.65]
Environmental noise	−7.21 [−23.51, 11.19]	3.26 [−15.08, 22.93]	−18.36 [−37.54, 0.83]	−6.18 [−21.47, 9.11]	−15.58 [−35.32, 5.02]	−24.99 [−45.46, −4.53]*

^*^p < 0.05, ^**^p < 0.01, ^***^p < 0.001. ^†^Indicates statistical significance after FDR correction.

**Figure 1:**
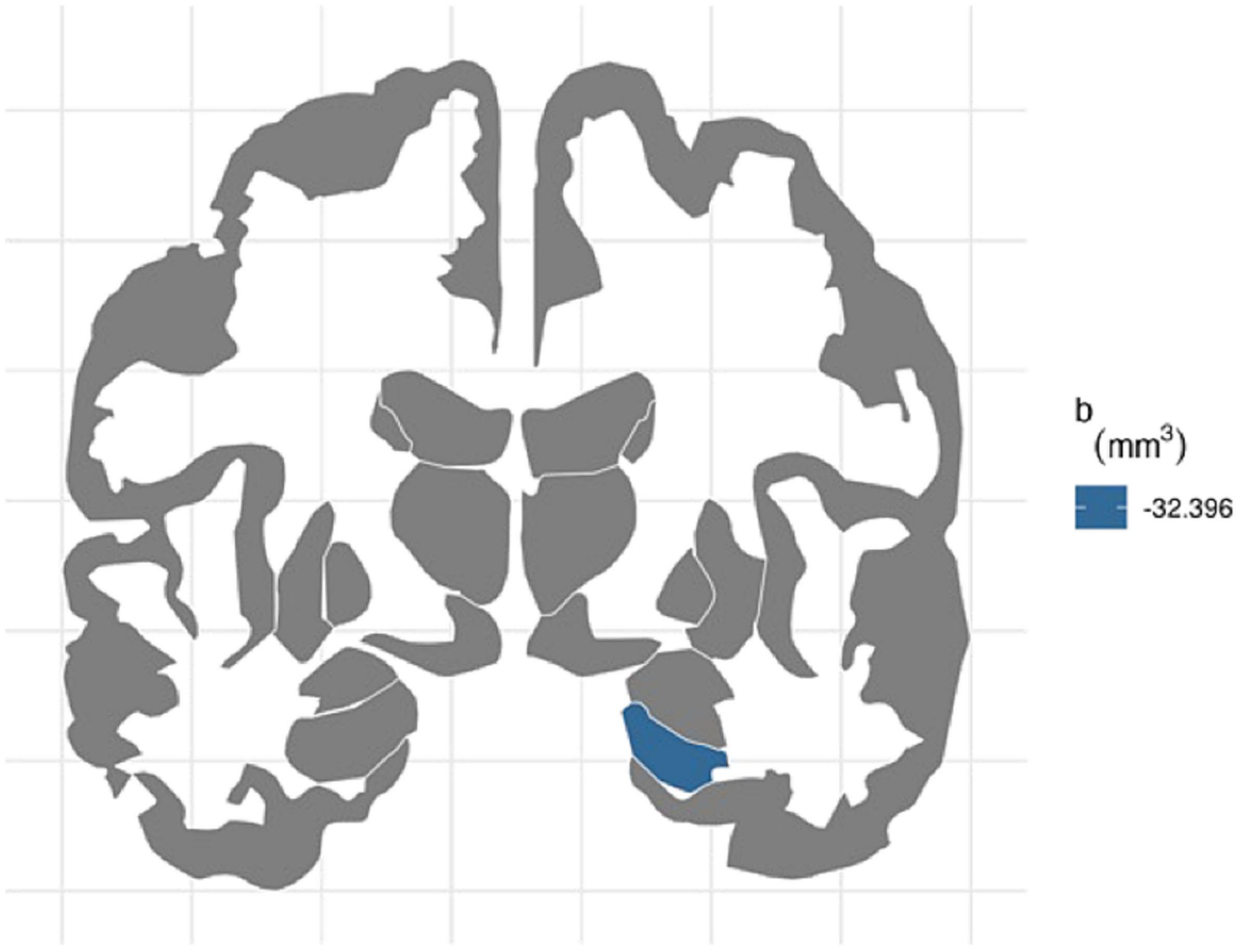
FDR-corrected association between ADI and brain volumes. area deprivation index was associated with smaller right hippocampal regions after FDR correction (b[95% CI] = −32.396[−48.962, −14.952]).

For the right hemisphere, ADI was associated with smaller volumes for entorhinal (b[95% CI] = −21.444[−37.606, −4.723], *p* < 0.05) and hippocampal regions (b[95% CI] = −32.396[−48.962, −14.952], *p* < 0.001), and ADI remained significant for hippocampal volume after FDR correction (Figure 1). More environmental noise was associated with smaller hippocampal volume (b[95% CI] = −24.985[−45.455, −4.532], *p* < 0.05), but this association did not remain significant after FDR correction.

### 3.3 Associations between prenatal alcohol and tobacco exposure and brain volumes

**Figure 2:**
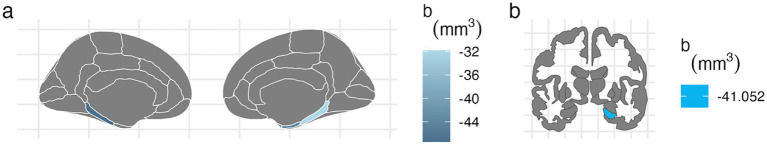
FDR-corrected associations between prenatal tobacco exposure and brain volumes. **(A)** PTE was associated with smaller volumes in the bilateral parahippocampal (left: b[95% CI] = −47.438[−80.758, −13.877]; right: b[95% CI] = −31.824[−59.901, −3.73]) and right entorhinal cortices (b[95% CI] = −43.149[−80.566, −5.77]) after FDR correction. **(B)** PTE was associated with smaller volume at the right hippocampal cortex (b[95% CI] = −41.052[−78.091, −4.04]) after FDR correction.

**Figure 3:**
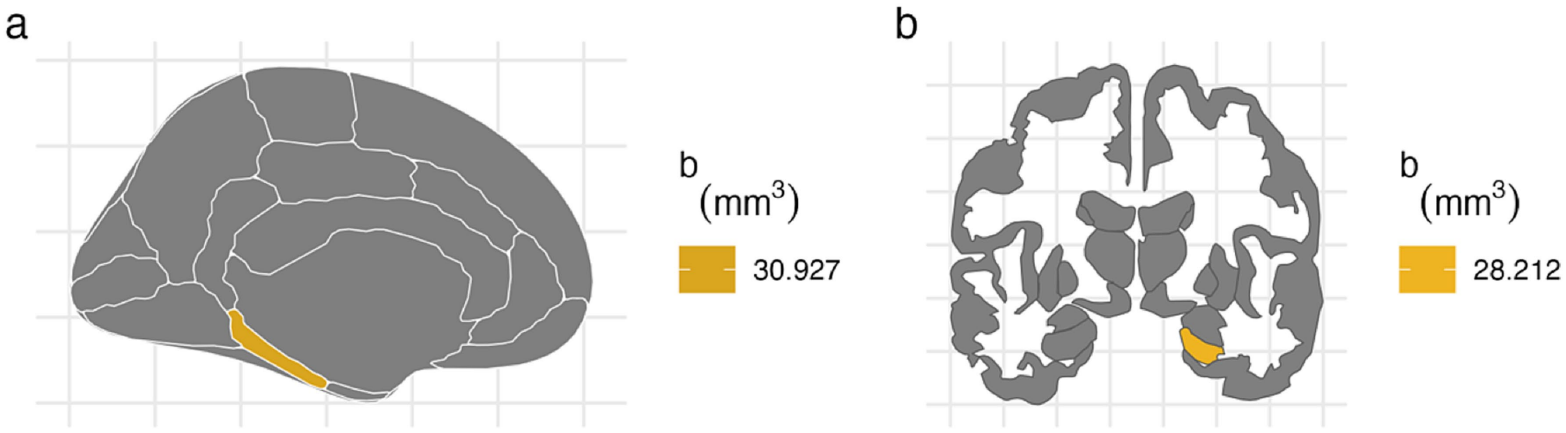
FDR-corrected associations between prenatal alcohol exposure and brain volumes. **(A)** PAE is associated with larger volumes at left parahippocampal cortex after FDR correction (b[95% CI] = 30.927[8.919, 53.275]). **(B)** PAE was associated with larger right hippocampal volume after FDR correction (b[95% CI] = 28.212[3.592, 52.776]).

For the left hemisphere (Table 5), PTE was associated with smaller volumes in parahippocampal (b[95% CI] = −47.438[−80.758, −13.877], *p* < 0.01), entorhinal (b[95% CI] = −42.612[−80.594, −4.771], *p* < 0.05), and hippocampal regions (b[95% CI] = −37.201[−72.415, −2.02], *p* < 0.05), but only the association with parahippocampal volume remained significant after FDR correction (Figure 2). PAE was associated with larger parahippocampal volume (b[95% CI] = 30.927[8.919, 53.275], *p* < 0.01), and this association remained significant after FDR correction (Figure 3).

**Table 5:** Estimate and 95% confidence intervals of prenatal exposure to alcohol and tobacco baseline, adjusting for age in months, sex assigned at birth, child race, ethnicity, whether the family is below federal poverty line, family education, and exposure to any substances prenatally other than alcohol or tobacco.

	Left hemisphere volumes			Right hemisphere volumes		
	Parahippocampal	Entorhinal	Hippocampal	Parahippocampal	Entorhinal	Hippocampal
PTE	−47.44 [−80.76, −13.88]**,^†^	−42.61 [−80.59, −4.77]*	−37.20 [−72.42, −2.02]*	−31.82 [−59.90, −3.73]*^,†^	−43.15 [−80.57, −5.77]*^, †^	−41.05 [−78.09, −4.04]*^, †^
PAE	30.93 [8.92, 53.28]**, ^†^	4.41 [−20.63, 29.69]	16.04 [−7.36, 39.38]	15.43 [−3.23, 34.07]	2.02 [−22.77, 26.89]	28.21 [3.59, 52.78]*^, †^

*p < 0.05, **p < 0.01, ***p < 0.001. ^†^Indicates statistical significance after FDR correction.

For the right hemisphere, PTE was associated with smaller volumes in parahippocampal (b[95% CI] = −31.824[−59.901, −3.73], *p* < 0.05), entorhinal (b[95% CI] = −43.149[−80.566, −5.77], *p* < 0.05), and hippocampal regions (b[95% CI] = −41.052[−78.091, −4.04], *p* < 0.05), and all three associations remained significant after FDR correction (Figure 2). PAE was associated with larger hippocampal volume (b[95% CI] = 28.212[3.592, 52.776], *p* < 0.05), and this association remained significant after FDR correction (Figure 3).

### 3.4 Associations between neighborhood environmental exposure and brain volumes stratified by prenatal alcohol exposure

In participants with PAE, for the left hemisphere, higher walkability index was associated with smaller parahippocampal volume (b[95% CI] = −25.41[−51.48, −1.55], *p* = 0.048; Table 6). However, this association did not remain significant after FDR correction.

**Table 6:** Estimates and 95% confidence intervals of neighborhood environment exposure in participants with prenatal alcohol exposure, adjusting for age in months, sex assigned at birth, child race, ethnicity, whether the family is below federal poverty line, family education, and exposure to any substances prenatally other than alcohol or tobacco.

	Left hemisphere volumes			Right hemisphere volumes		
	Parahippocampal	Entorhinal	Hippocampal	Parahippocampal	Entorhinal	Hippocampal
Proximity to major roads	−0.96 [−19.23, 17.52]	−3.3 [−24.1, 18.25]	−0.23 [−20.04, 18.9]	2.56 [−13.35, 18.09]	−4.63 [−25.07, 15.56]	−10.33 [−30.66, 9.64]
National walkability index	−25.41 [−51.48, −1.55]*	−2.59 [−34.09, 23.25]	14.83 [−11.65, 42.02]	9.1 [−12.57, 29.85]	−4.37 [−31.88, 23.73]	20.26 [−7.35, 49]
Traffic count	−20.7 [−46.06, 4.46]	18.22 [−11.39, 46.22]	3.13 [−23.6, 30.48]	−17.5 [−39.24, 4.03]	18.41 [−10.15, 46.23]	−2.1 [−29.81, 26.64]
No_2_	−0.5 [−25.71, 23.19]	8.12 [−17.07, 33.77]	0.04 [−28.65, 28.04]	3.76 [−17.18, 24.58]	5.6 [−22.68, 34.58]	2.11 [−28.59, 32.35]
Pm_2.5_	−18.6 [−43.72, 7.33]	−24.17 [−50.43, 7.1]	−8.83 [−38.01, 20.37]	−17.22 [−39.78, 4.48]	−34.03 [−63.52, −1.83]*	−9.87 [−41.3, 21.09]
O_3_	3.66 [−17.52, 23.38]	−5.63 [−29.73, 15.88]	−5.58 [−27.48, 17.43]	8.59 [−8.35, 26.94]	−10.57 [−33.13, 13.4]	−14.91 [−37.98, 9.96]
Lead exposure	11.95 [−9.55, 34.33]	15.05 [−9.31, 40.05]	−2.46 [−25.84, 21.28]	3.06 [−15.28, 22.42]	32.09 [7.28, 56.34]*	−2.98 [−27.71, 21.44]
Area deprivation index	−17.67 [−42.68, 9.85]	−8.07 [−35.05, 21.62]	−23.4 [−51.8, 7.46]	−9.78 [−31.43, 14.83]	−16.24 [−45.33, 14.52]	−32.86 [−63.02, −0.48]*
Parent-report neighborhood safety	−15.53 [−38.29, 7.45]	1.48 [−24.37, 28.33]	8.11 [−16.26, 32.06]	−7.9 [−27.48, 11.62]	2.16 [−23.3, 27.25]	8.67 [−16.36, 33.67]
Environmental noise	17.42 [−13.18, 48.68]	8.93 [−23.84, 42.82]	1.62 [−34.95, 36.11]	3.8 [−23.17, 30.31]	−2.14 [−38.5, 33.34]	−8.38 [−46.54, 28.33]

*p < 0.05, **p < 0.01, ***p < 0.001. ^†^Indicates statistical significance after FDR correction.

**Figure 4:**
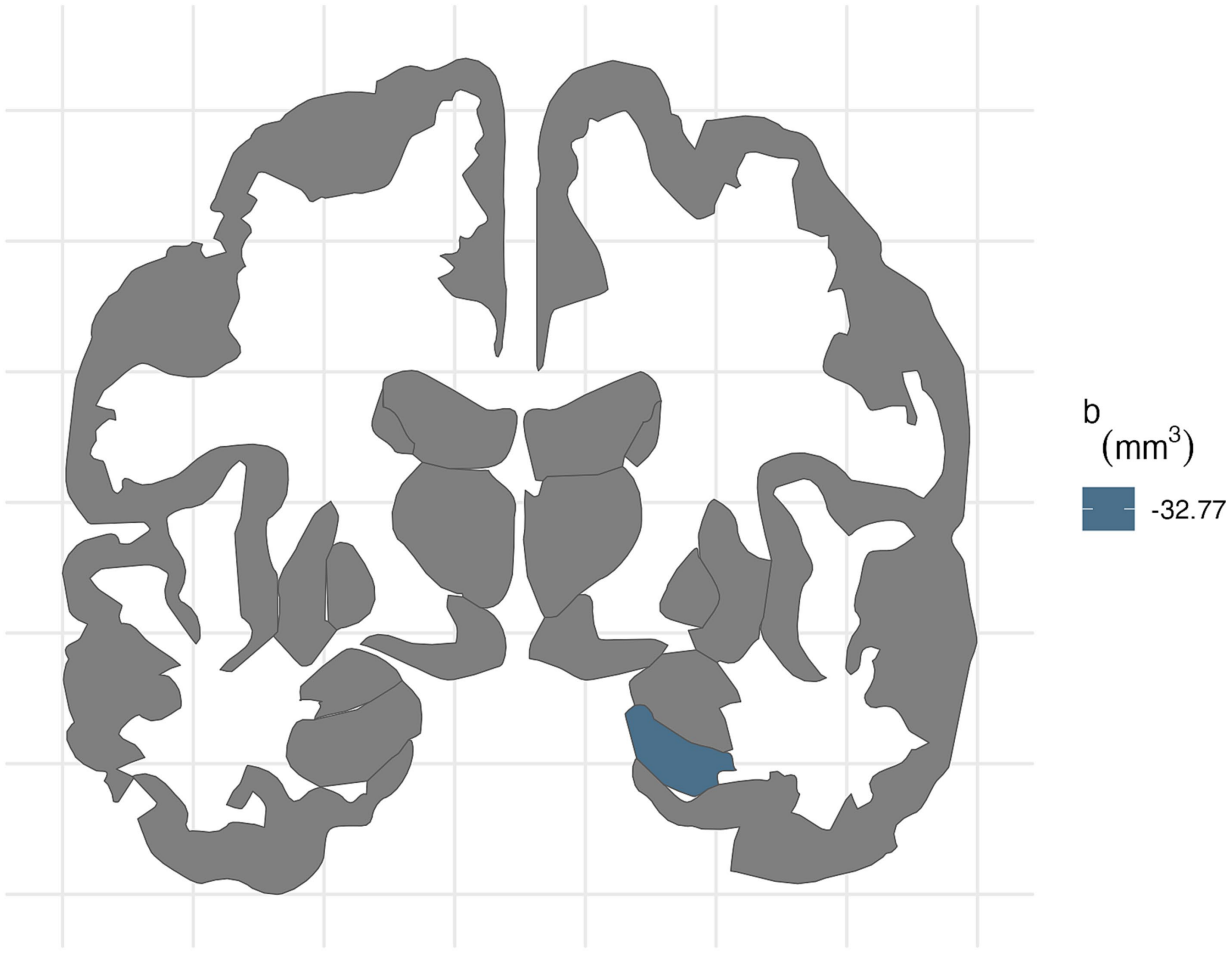
FDR-corrected associations between neighborhood environmental exposures and brain volumes stratified by prenatal tobacco exposure. In participants without PTE, ADI was associated with smaller right hippocampal volume (b[95% CI] = −32.77[−50.8, −13.79]) after FDR correction.

In participants with PAE, for the right hemisphere, higher average levels of PM_2.5_ was associated with smaller entorhinal volumes (b[95% CI] = −34.03[−63.52, −1.83], *p* = 0.03; Table 6). Higher lead exposure was associated with larger entorhinal volumes (b[95% CI] = 32.09[7.28, 56.34], *p* = 0.01). More area deprivation was associated with smaller hippocampal volume (b[95% CI] = −32.86[−63.02, −0.48], *p* = 0.039). None of these associations retained significance after FDR correction (Figure 4).

In participants without PAE, more area deprivation was associated with smaller left entorhinal volume (b[95% CI] = −21.71[−40.64, −3.33], *p* = 0.03) and more environment noise was associated with lower hippocampal volume (b[95% CI] = −27.28[−49.27, −4.03], *p* = 0.02; Table 7). However, these associations did not remain significant after FDR correction. For the right hemisphere, more area deprivation was associated with smaller entorhinal (b[95% CI] = −25.33[−44.9, −5.37], *p* = 0.01) and hippocampal volume (b[95% CI] = −25.39[−44.77, −4.91], *p* = 0.01). More environmental noise was associated with smaller hippocampal volume (b[95% CI] = −35.12[−58.79, −10.58], *p* = 0.005). None of these associations retained statistical significance after FDR correction.

**Table 7:** Estimates and 95% confidence intervals of neighborhood environment exposure in participants without prenatal alcohol exposure, adjusting for age in months, sex assigned at birth, child race, ethnicity, whether the family is below federal poverty line, family education, and exposure to any substances prenatally other than alcohol or tobacco.

	Left hemisphere volumes			Right hemisphere volumes		
	Parahippocampal	Entorhinal	Hippocampal	Parahippocampal	Entorhinal	Hippocampal
Proximity to major roads	−5.97 [−17.83, 8.06]	1.39 [−12.62, 16.5]	2.35 [−11.11, 15.9]	−10.71 [−21.38, 0.27]	2.58 [−11.68, 17.32]	9.10 [−5.07, 23.4]
National walkability index	5.85 [−11.55, 20.11]	5.36 [−12.13, 24.51]	5.09 [−12.16, 22.47]	5.31 [−8.65, 19.16]	7.31 [−11.14, 26.06]	8.30 [−9.83, 27.12]
Traffic count	0.85 [−12.11, 13.24]	0.88 [−14.48, 14.99]	0.94 [−12.88, 14.52]	1.58 [−9.36, 12.55]	4.87 [−9.99, 19.38]	4.39 [−10.08, 18.81]
No_2_	−13.54 [−28.33, −0.03]	−8.79 [−26.43, 8.96]	5.16 [−12.86, 23.24]	−1.43 [−16.47, 13.15]	14.14 [−5.4, 33.64]	10.39 [−9.3, 29.86]
Pm_2.5_	10.42 [−5.21, 25.32]	13.24 [−5.42, 31.96]	14.32 [−4.95, 32.84]	−3.57 [−18.65, 11.99]	2.02 [−18.65, 22.05]	10.34 [−9.99, 30.48]
O_3_	0.81 [−13.2, 12.32]	−12.59 [−28.98, 1.51]	12.16 [−1.87, 26.39]	5.92 [−5.32, 17.5]	−1.41 [−16.87, 13.57]	8.37 [−6.54, 23.61]
Lead exposure	−1.8 [−15.46, 15.58]	9.84 [−7.87, 26.87]	8.05 [−8.48, 24.86]	−1.05 [−14.23, 12.78]	2.57 [−15.48, 20.32]	15.01 [−2.92, 32.55]
Area deprivation index	−1.39 [−16.98, 13.77]	−21.71 [−40.64, −3.33]*	−16.6 [−34.64, 2.33]	−3.38 [−18.01, 11.8]	−25.33 [−44.9, −5.37]*	−25.39 [−44.77, −4.91]*
Parent-report neighborhood safety	−6.15 [−19.94, 8.92]	3.35 [−12.94, 19.74]	−4.85 [−19.93, 10.59]	−8.02 [−20.07, 4.33]	−5.98 [−22.12, 10.54]	−3.59 [−19.49, 12.65]
Environmental noise	−8.3 [−25.86, 14.46]	3.94 [−17.99, 27.75]	−27.28 [−49.27, −4.03]*	−14.54 [−32.42, 3.81]	−19.31 [−43.1, 6.83]	−35.12 [−58.79, −10.58]**

*p < 0.05, **p < 0.01, ***p < 0.001. ^†^Indicates statistical significance after FDR correction.

### 3.5 Associations between neighborhood environmental exposure and brain volumes stratified by prenatal tobacco exposure

For the left hemisphere (Table 8), in participants with PTE, more environmental noise was associated with larger hippocampal volume (b[95% CI] = 61.15[12.22,110.08], *p* = 0.01). This association did not retain statistical significance after FDR correction. For the right hemisphere, in participants with PTE, higher average O_3_ level was associated with larger entorhinal volume (b[95% CI] = 43.78[5.96, 81.59], *p* = 0.02; Table 9). This association did not retain statistical significance after FDR correction.

**Table 8:** Estimates and 95% confidence intervals of neighborhood environment exposure in participants with prenatal tobacco exposure, adjusting for age in months, sex assigned at birth, child race, ethnicity, whether the family is below federal poverty line, family education, and exposure to any substances prenatally other than alcohol or tobacco.

	Left hemisphere volumes			Right hemisphere volumes		
	Parahippocampal	Entorhinal	Hippocampal	Parahippocampal	Entorhinal	Hippocampal
Proximity to major roads	15.25 [−13.03, 44.78]	−10.86 [−45.35, 23.63]	−6.58 [−38.29, 25.13]	10.34 [−14.04, 36.46]	4.44 [−29.28, 38.17]	−21.21 [−53.63, 11.21]
National walkability index	−20.27 [−62.7, 14.23]	−34.68 [−79.83, 10.48]	−9.52 [−51.04, 32]	3.39 [−33.26, 33.57]	−39.55 [−83.71, 4.61]	−0.03 [−42.48, 42.42]
Traffic count	5.3 [−32.84, 41.28]	−29.62 [−73.84, 14.6]	−2.62 [−43.28, 38.03]	−25.95 [−58.01, 6.73]	−23.48 [−66.72, 19.76]	−2.95 [−44.52, 38.62]
NO_2_	5.63 [−29.58, 38.85]	13.86 [−26.9, 54.63]	36.74 [−0.74, 74.22]	14.41 [−14.25, 45.54]	33.73 [−6.13, 73.6]	31.89 [−6.44, 70.22]
PM_2.5_	9.24 [−27.26, 42.51]	−2.78 [−44.28, 38.71]	−0.05 [−38.21, 38.1]	−4.16 [−36.95, 24.26]	16.51 [−24.07, 57.09]	26.03 [−12.98, 65.04]
O_3_	9.39 [−27.29, 38.83]	7.45 [−31.22, 46.12]	−5.32 [−40.87, 30.23]	22.28 [−5.94, 50.68]	43.78 [5.96, 81.59]*	6.23 [−30.12, 42.58]
Lead exposure	16.84 [−16.05, 52.31]	−1.69 [−42.48, 39.1]	−29.3 [−66.8, 8.2]	−3.59 [−33.74, 25.98]	−2.44 [−42.33, 37.44]	−28.74 [−67.08, 9.61]
Area deprivation index	−18.01 [−52.7, 16.12]	7.27 [−33.79, 48.32]	2.35 [−35.4, 40.09]	−3.74 [−32.22, 27.91]	15.5 [−24.65, 55.65]	−4.37 [−42.97, 34.22]
Parent-report neighborhood safety	−6.21 [−36.65, 24.84]	1.58 [−35.1, 38.26]	7.95 [−25.78, 41.67]	7.13 [−19.43, 34.27]	7.94 [−27.93, 43.81]	4.98 [−29.5, 39.47]
Environmental noise	10.48 [−27.38, 58.26]	61.15 [12.22, 110.08]*	−5.02 [−50.01, 39.97]	15.61 [−17.99, 53.84]	28.17 [−19.68, 76.02]	−36.76 [−82.77, 9.24]

*p < 0.05, **p < 0.01, ***p < 0.001. ^†^Indicates statistical significance after FDR correction.

**Table 9:** Estimates and 95% confidence intervals of neighborhood environment exposure in participants without prenatal tobacco exposure, adjusting for age in months, sex assigned at birth, child race, ethnicity, whether the family is below federal poverty line, family education, and exposure to any substances prenatally other than alcohol or tobacco.

	Left hemisphere volumes			Right hemisphere volumes		
	Parahippocampal	Entorhinal	Hippocampal	Parahippocampal	Entorhinal	Hippocampal
Proximity to major roads	−8.77 [−19.5, 2.83]	1.87 [−10.4, 14.84]	3.18 [−8.54, 14.86]	−9.08 [−18.44, 0.32]	−1.17 [−13.53, 11.46]	6.01 [−6.33, 18.33]
National walkability index	1.14 [−13.67, 14.53]	6.68 [−8.94, 22.5]	7.4 [−7.73, 22.79]	5.3 [−6.93, 17.53]	6.12 [−9.84, 22.66]	10.3 [−5.71, 26.71]
Traffic count	−5.24 [−17.27, 6.35]	6 [−8.19, 18.96]	2.64 [−9.96, 15.18]	1.41 [−8.69, 11.47]	10.18 [−3.37, 23.45]	4.65 [−8.59, 17.94]
NO_2_	−10.1 [−24.79, 3.2]	−5.01 [−20.4, 10.35]	2.03 [−14.81, 18.62]	−1.37 [−15.15, 12.02]	7.06 [−10.27, 24.36]	8.09 [−10.32, 26.16]
PM_2.5_	3.26 [−11.1, 17.9]	3.91 [−12.39, 20.78]	10.7 [−6.75, 27.97]	−6.39 [−20.32, 7.66]	−8.44 [−26.6, 9.61]	2.8 [−16.01, 21.53]
O_3_	4.17 [−8.55, 15.06]	−12.28 [−26.22, 0.16]	8.35 [−4.21, 21.12]	3.64 [−6.42, 13.99]	−8.22 [−21.53, 5.18]	1.27 [−12.11, 14.92]
Lead exposure	3.03 [−9.5, 17.35]	9.07 [−5.78, 23.76]	10.7 [−3.49, 25.1]	1.92 [−9.41, 13.69]	13.92 [−1.4, 28.95]	14.21 [−0.96, 29.33]
Area deprivation index	−5.96 [−20.34, 9.19]	−20.82 [−37.41, −3.97]*	−23.29 [−40.08, −5.37]**	−12.99 [−26.54, 1.55]	−26.76 [−44.37, −8.47]**	−32.77 [−50.8, −13.79]*** ^†^
Parent-report neighborhood safety	−7.22 [−19.77, 6.28]	5.04 [−9.38, 19.98]	−5.48 [−18.97, 8.23]	−9.11 [−19.89, 1.93]	−3.64 [−18.06, 10.96]	−4.91 [−19.13, 9.5]
Environmental noise	−8.24 [−25.33, 11.25]	−1.36 [−20.85, 19.48]	−21.9 [−42.43, −1.37]*	−9.32 [−25.84, 7.2]	−19.48 [−40.79, 2.69]	−26.07 [−48.12, −4.04]*

*p < 0.05, **p < 0.01, ***p < 0.001. ^†^Indicates statistical significance after FDR correction.

For the left hemisphere (Table 9), in participants without PTE, higher area deprivation was associated with smaller volumes in entorhinal (b[95% CI] = −20.82[−37.41, −3.97], *p* = 0.018) and hippocampal regions (b[95% CI] = −23.29[−40.08, −5.37], *p* = 0.008). Higher exposure to environmental noise was associated with smaller hippocampal volume (b[95% CI] = −21.9[−42.43, −1.37], *p* = 0.039). These associations did not retain significance after FDR correction.

For the right hemisphere, in participants without PTE, higher area deprivation was significantly associated with smaller volumes in the entorhinal (b[95% CI] = −26.76[−44.37, −8.47], *p* = 0.004) and hippocampal area (b[95% CI] = −32.77[−50.8, −13.79], *p* < 0.001). The association between area deprivation and hippocampus remained significant after FDR corrections. Higher environment noise (b[95% CI] = −26.07[−48.12, −4.04], *p* = 0.02) were associated with smaller hippocampal volume, but this association did not remain significant after FDR corrections.

## 4 Discussion

Our analysis found that neighborhood environmental exposure at age 9–11 years was associated with differences in brain volumes after adjusting for individual-level socioeconomic status indicators. Specifically, higher area deprivation was associated with smaller right hippocampal volume. PAE was associated with larger volumes in left parahippocampal and hippocampal regions, while PTE was associated with smaller volumes in bilateral parahippocampal, right entorhinal, and right hippocampal regions. Moreover, PTE modified the relationship between neighborhood environment and brain volumes. For children with PTE, none of the associations between neighborhood environment and brain volumes remained significant after corrections for multiple comparison. Among children without PTE, higher area deprivation was associated with smaller right hippocampal volumes. When we stratified by PAE, neither PAE nor no PAE groups showed significant associations between neighborhood environment and brain volumes after corrections for multiple comparison.

We found that PAE was associated with larger volumes in parahippocampal and hippocampal regions. While this finding is similar to studies based on the same cohort (Lees et al., 2020), previous studies observed smaller volumes at the hippocampal regions comparing children or adolescents exposed to alcohol prenatally to their non-exposed peers (Willoughby et al., 2008; Nardelli et al., 2011; Treit et al., 2013). Most of these studies examined subjects with severe prenatal alcohol exposure who qualified for a clinical diagnosis. In many cases, prenatal alcohol exposure history was ascertained by adoption or foster records that substantiated the child’s removal due to the biological mother’s alcohol misuse or heavy drinking in pregnancy (Willoughby et al., 2008). Participants analyzed in the current study were typically developed children with low to moderate exposure and without any clear neurological disorders. Prenatal exposure to alcohol was reported by biological mothers and mostly before pregnancy recognition. This difference in the dosage and timing of the exposure may have contributed to the different directionality in findings. Regarding the timing of the exposure, preclinical and human neuroimaging studies showed that the developing brain may be more sensitive to sustained PAE throughout gestation and PAE during the third trimester, in comparison to first or second trimester exposure (Maier et al., 1999; Thompson et al., 2024; Ramadoss et al., 2007; Liang et al., 2024). Since the PAE group in our study includes both exposure before pregnancy recognition and after pregnancy recognition, it is possible that sustained exposure throughout pregnancy and PAE during late pregnancy may have contributed to our finding. As we are limited by the insufficient information on exposure timing, further investigation is needed to elucidate how the effect of low to moderate PAE in early stage of gestation may differ from those in later gestation stage. Regarding dosage dependent response to PAE, low to moderate levels of PAE have been consistently associated with changes in brain development through epigenetic, cellular and intracellular mechanisms, specifically in the hippocampus in preclinical rodent models (Livy et al., 2003; Comasco et al., 2018). While it is clear that the effect of PAE on human development can differ by dosage (Coles et al., 2015), a recent narrative review and a meta-analysis concluded that further investigation was needed on the effect of low to moderate PAE due to methodological issues and inconsistent directionality of findings (Comasco et al., 2018; Flak et al., 2014). Pooling across cohort studies, Flak et al. (2014) observed a small but significant positive association between mild to moderate PAE and child cognition, although this association did not remain significant after the post-hoc exclusion of one large study or when only moderate PAE was included. In the same meta-analysis, moderate PAE was significantly associated with child behavior, while mild PAE was not, which suggests a dose–response relationship. Given that 19.6% of pregnant persons in their first trimester reported at least one drink in the past month (England et al., 2020), it is likely that a sizable portion of pregnancies are impacted by low to moderate PAE. Further examination is needed regarding the effect of low-to-moderate level PAE on a population level.

In the current analysis, PTE was associated with smaller parahippocampal, hippocampal, and entorhinal volumes. Previous studies on children of similar age range have mixed findings. Findings from the Generation R study based in Netherlands consistently showed no differences in parahippocampal, hippocampal, and entorhinal regions between children who were exposed to tobacco prenatally and children who were not (Zou et al., 2022; El Marroun et al., 2014; El Marroun et al., 2016). In contrast, Marshall et al. (2022) reported that PTE was associated with smaller left entorhinal and right hippocampal volume in a South Africa prospective cohort, although these associations did not pass FDR corrections. One possible reason for the mixed findings is that both the current study and the study by Marshall et al. (2022) adjusted for PAE while examining the associations with PTE, while the analysis from the Generation R study adjusted for other exposure such as cannabis but not alcohol specifically. Further research is needed to examine how the associations between prenatal exposure to tobacco and brain structure may change in the presence of other co-exposures.

We found that neighborhood area deprivation was negatively associated with hippocampal volume. Moreover, it is important to highlight that this association holds after accounting for individual/family-level socioeconomic characteristics. Previous studies have shown that neighborhood-level socioeconomic status is associated with differences in structural brain measures. Gur et al. (2019) found that census-tract level neighborhood socioeconomic composite score was associated with differences in brain volume and gray matter density. Neighborhood poverty was associated with smaller brain volumes in regions including the hippocampus (Taylor et al., 2020). Whittle et al. (2017) showed that longitudinally, children living in neighborhoods with higher socioeconomic disadvantage had relatively increased cortical thickening over time. Interestingly, although various built and social environment measures were included in the analysis, none of these other measures showed significant associations with the brain volumes. We would expect differential effects of neighborhood environment factors on brain regional structural development, given the different hypothesized underlying mechanisms. More institutional resources, better infrastructure and safer neighborhoods may lead to a more enriched environment for children who live in these neighborhoods. Preclinical models demonstrated that environmental enrichment can enhance cognition through anatomical changes in the brain, including increased cortical thickness, hippocampal neurogenesis, and volumetric changes in hippocampus and entorhinal cortex (Sale et al., 2009; Scholz et al., 2015; Hirase and Shinohara, 2014). Evidence supports that environmental enrichment can modify genetic expressions linked to neuronal structure, synaptic plasticity, and neuronal excitability and affect cholinergic, serotoninergic, and noradrenergic systems (Sale et al., 2009). In contrast, traffic-related air pollution may affect brain development by crossing the blood–brain barrier and inducing an immune response that contributes to widespread neuroinflammation in the brain. However, although the hypothesized pathways may be different, it is difficult to isolate the contribution of various neighborhood environment factors due to clustering of risk factors (Brockmeyer and D’Angiulli, 2016). For instance, it is possible that area deprivation index, as a composite measure, reflects a combination of neighborhood environment factors. Environmental inequality in the US has been well documented (Salazar et al., 2019). Areas with higher socioeconomic deprivation are more likely to be exposed to hazardous air pollutants (Young et al., 2012). Future studies may disentangle the pathways through case–control or natural experiment study designs that aim to isolate one aspect of neighborhood environment.

Our findings did not support a disadvantage hypothesis in relation to PTE or PAE. We found that for children without PTE, living in a high deprivation neighborhood was associated with smaller brain volumes, but this relationship did not hold for children with PTE. This finding is in contrast with previous studies that showed worse cognitive and behavioral outcomes among children and adolescents who experienced PTE and other postnatal adversities in comparison to children and adolescents who only experienced PTE. Rauh (2004) found that children with prenatal environmental tobacco exposure had worse cognition at age two, and material hardship exacerbated the developmental delay in a sample of urban minority women. Another prospective study found that children with prenatal exposure to tobacco and stress in conjunction with high postnatal stress exhibited worst executive control and more disruptive behavior (Clark et al., 2016). One possible explanation for this contrasting finding is that participants with PTE may be more likely to have been exposed to adversities than participants without PTE. Previous literature has shown that living in neighborhoods with lower median household income, higher poverty rate, lower percentage of residents with at least a college degree, and higher percentage of uninsured residents was associated with higher odds of smoking while pregnant. Within our study sample, children with PTE were more likely to have lower family income and parental education than children without PTE (Galiatsatos et al., 2020). It is possible that because children with PTE were more likely to experience social adversities in general, the association between residential neighborhood deprivation and brain was not easily distinguishable from the effects of other cumulative adversities. Alternatively, it is also possible that PTE has altered the postnatal environmental sensitivity, so that children with PTE are not as responsive to environmental influence as children without PTE. Further research is needed in comparing children with PTE to children without PTE who live in similar neighborhoods to examine whether postnatal environmental sensitivity is different following PTE.

We did not observe significant associations between neighborhood environment and brain volumes after stratifying by PAE. It is possible that stratification may have impacted the power of the analysis. The associations between area deprivation and brain volumes were significant in the unexposed group before FDR correction, which resembles the findings of the total sample. While the limited number of prior studies on neighborhood environment and the brain and the inconsistency of how neighborhood environment was measured made it difficult to determine the expected effect size with high confidence, the existing studies on area deprivation and structural brain development suggest a significant, albeit small effect (Taylor et al., 2020). A recent study on air pollution, physical activity and brain volume also showed a significant but small effect between air pollution and grey matter volume (absolute values of standardized coefficient ranging between 0.029 and 0.042) (Furlong et al., 2022). To detect an interaction of such small effect may require more power. Further research is needed to examine whether the effect of neighborhood environment on brain differs by PAE.

There are a few limitations of this study. First, prenatal substance exposures were collected retrospectively. These measures may be subject to recall bias due to the stigma associated with substance use during pregnancy. However, the questionnaire separated substance use before pregnancy recognition and after pregnancy recognition. Mothers may feel less stigmatized about reporting legal substance use before they even recognized that they were pregnant. The prevalence of alcohol use during pregnancy reported in the current study sample is comparable to the prevalence of pregnant respondents who endorse alcohol use during pregnancy in national surveys (England et al., 2020). Second, the analysis does not consider the duration of exposure. The association between neighborhood environment and brain may differ by the duration of residency in a neighborhood. Third, the current study is cross-sectional. Brain development is dynamic, especially at the on-set of puberty. The association between neighborhood environment, prenatal exposure to substances, and brain may change during puberty. Lastly, while we focused on three regions of interest (hippocampus, parahippocampal and entorhinal regions), it is possible that other regions of the brain are also affected by neighborhood environment, PAE and PTE. For instance, amygdala-hippocampus circuit plays an important role in stress response and studies have shown volumetric changes in the amygdala following institutional rearing (Tottenham, 2009; Tottenham et al., 2010).

To our best knowledge, our study is the first to examine whether prenatal exposure to alcohol or tobacco modify the association between neighborhood environment and structural brain development in children. We used a nationally representative sample of children across the US with neighborhood environment measures that encompassed social, built, and institutional environments. Our findings supported that neighborhood environment was associated with brain volumes at age 9–11 even after adjusting for individual-level socioeconomic status and this association differed by prenatal exposure to tobacco. Future research on the effect of prenatal exposure to substances on brain development may need to consider how postnatal neighborhood environment may modify this relationship.

## Data Availability

Publicly available datasets were analyzed in this study. The data is available on the National Institute of Mental Health Data Archive (https://nda.nih.gov/study.html?id=2147).

## Footnotes

^1^ https://abcdstudy.org/scientists/data-sharing/

^2^ https://abcdstudy.org/about/
