# Supplementary material for: The association between neighborhood environment, prenatal exposure to alcohol and tobacco, and structural brain development
Source: Front Hum Neurosci. 2025 Feb 18;19:1531803. doi: 10.3389/fnhum.2025.1531803 (PMC11876420; doi:10.3389/fnhum.2025.1531803)
Supplement: Supplementary file 1 [file Table_1.docx]

Supplementary Material

# Supplementary Tables

## Supplementary Table 1, Prenatal exposure to other substances by prenatal alcohol exposure and prenatal tobacco exposure.

|  | | **PAE** | | | | **PTE** | | | | **Total**  (n = 7887) | |
| --- | --- | --- | --- | --- | --- | --- | --- | --- | --- | --- | --- |
|  |  | ***No Exposure***  (n = 5384) | | ***Exposed***  (n = 2041) | | ***No Exposure***  (n = 6612) | | ***Exposed***  (n = 1063) | |  |  |
|  |  | N | % | N | % | N | % | N | % | N | % |
| **Marijuana** |  | 173 | 3.21 | 291 | 14.26 | 178 | 2.69 | 286 | 26.90 | 482 | 6.11 |
|  | missing | 7 | 0.13 | 51 | 2.50 | 33 | 0.50 | 50 | 4.70 | 235 | 2.98 |
| **Crack/Cocaine** |  | 7 | 0.13 | 48 | 2.35 | 8 | 0.12 | 43 | 4.05 | 65 | 0.82 |
|  | missing | 3 | 0.06 | 47 | 2.30 | 6 | 0.09 | 52 | 4.89 | 206 | 2.61 |
| **Heroine/Morphine** |  | 5 | 0.09 | 13 | 0.64 | 6 | 0.09 | 11 | 1.03 | 20 | 0.25 |
|  | missing | 3 | 0.06 | 57 | 2.79 | 5 | 0.08 | 60 | 5.64 | 220 | 2.79 |
| **OxyContin** |  | 10 | 0.19 | 21 | 1.03 | 9 | 0.14 | 23 | 2.16 | 33 | 0.42 |
|  | missing | 9 | 0.17 | 59 | 2.89 | 12 | 0.18 | 61 | 5.74 | 231 | 2.93 |
| **Other substances** |  | 29 | 0.54 | 46 | 2.25 | 34 | 0.51 | 38 | 3.57 | 78 | 0.99 |
|  | missing | 36 | 0.67 | 78 | 3.82 | 46 | 0.70 | 84 | 7.90 | 288 | 3.65 |

*Note: Each substance exposure category combined participants whose caregiver endorsed substance use of this category before or after pregnancy recognition. The percentage columns are calculated by exposure group (PAE, no PAE, PTE, no PTE, and total sample).*

## Supplementary Table 2, Average Frequency of Prenatal exposure to other substances by prenatal alcohol exposure and prenatal tobacco exposure. The average frequency is calculated for times per day.

|  |  | **PAE** | | **PTE** | |
| --- | --- | --- | --- | --- | --- |
|  |  | **No exposure** | **Exposed** | **No exposure** | **Exposed** |
| **Marijuana Before Pregnancy Recognition** | Mean (SD) | 2.47(3.53) | 1.94(2.11) | 1.79(1.33) | 2.44(3.49) |
|  | n | 171 | 288 | 178 | 281 |
|  | Missing | 29 | 98 | 36 | 88 |
| **Marijuana After Pregnancy Recognition** | Mean (SD) | 2.24(1.51) | 1.73(1.20) | 2.08(1.42) | 1.88(1.32) |
|  | n | 58 | 108 | 51 | 112 |
|  | Missing | 13 | 54 | 11 | 52 |
| **Cocaine/Crack Before Pregnancy Recognition** | Mean (SD) | 3.2(2.49) | 2.0(1.32) | 3.57(2.00) | 2.07(1.54) |
|  | n | 7 | 46 | 8 | 42 |
|  | Missing | 2 | 30 | 1 | 28 |
| **Cocaine/Crack After Pregnancy Recognition** | Mean (SD) | - | 2(2) | 3(2.83) | 6 |
|  | n | 1 | 20 | 2 | 19 |
|  | Missing | 1 | 24 | 0 | 18 |
| **Heroine/Morphine Before Pregnancy Recognition** | Mean (SD) | 3(1.41) | - | 2 | 4 |
|  | n | 3 | 12 | 3 | 11 |
|  | Missing | 1 | 12 | 2 | 10 |
| **Heroine/Morphine After Pregnancy Recognition** | Mean (SD) | 3(1.41) | - | 1(1.41) | 4 |
|  | n | 4 | 7 | 5 | 6 |
|  | Missing | 2 | 7 | 3 | 5 |
| **OxyContin Before Pregnancy Recognition** | Mean (SD) | 2.14(1.35) | 2.75(1.72) | 2.0(1.41) | 2.5(1.20) |
|  | n | 10 | 18 | 8 | 22 |
|  | Missing | 3 | 14 | 4 | 14 |
| **OxyContin After Pregnancy Recognition** | Mean (SD) | 2.0(1.14) | 3.5(0.7) | 3 | 2.67(1.53) |
|  | n | 4 | 13 | 4 | 12 |
|  | Missing | 2 | 11 | 3 | 9 |

## Supplementary Table 3, Coefficients and 95% CI of exposure to any substances prenatally other than alcohol or tobacco (not FDR corrected).

|  | **Parahippocampal Volume** | | **Entorhinal Volume** | | **Hippocampal Volume** | |
| --- | --- | --- | --- | --- | --- | --- |
|  | b[95%CI] | *P* | b[95%CI] | *P* | b[95%CI] | *P* |
| **Left Hemisphere- Neighborhood environment** | -25.39[-65.4,14.48] | 0.21 | 2.67[-42.57,48.1] | 0.91 | -3.98[-45.78,37.68] | 0.85 |
| **Right Hemisphere- Neighborhood environment** | -7.61[-41.18,25.8] | 0.66 | -0.52[-45.14,43.88] | 0.98 | -8.39[-52.2,35.31] | 0.71 |
| **Left Hemisphere-**  **PAE and PTE** | -3.03[-46.24,39.97] | 0.89 | 19.45[-29.46,68.2] | 0.44 | 7.16[-38.06,52.5] | 0.76 |
| **Right Hemisphere –**  **PAE and PTE** | 5.01[-31.09,41.16] | 0.79 | 18.42[-29.74,66.51] | 0.45 | 3.56[-44.01,51.22] | 0.88 |
| **Left Hemisphere-**  **Stratified PAE** | -28.34[-81.87,23.8] | 0.3 | -16.39[-77.81,44.5] | 0.6 | 2.44[-53.32,57.43] | 0.93 |
| **Right Hemisphere-**  **Stratified PAE** | 6.65[-39.2,51.08] | 0.77 | -8.27[-66.72,49.4] | 0.78 | 4.08[-53.62,60.92] | 0.89 |
| **Left Hemisphere-**  **Stratified No PAE** | -55.09[-124.76,14.58] | 0.12 | 40.64[-38.15,119.43] | 0.31 | -14.1[-87.07,58.88] | 0.7 |
| **Right Hemisphere –**  **Stratified No PAE** | -43.59[-101.8,14.62] | 0.14 | 17.64[-60.39,95.68] | 0.66 | -37.94[-114.61,38.73] | 0.33 |
| **Left Hemisphere- Stratified PTE** | 0.93[-61.57,63.43] | 0.98 | 56.76[-16.39,129.91] | 0.13 | 40.03[-27.23,107.28] | 0.24 |
| **Right Hemisphere- Stratified PTE** | -29.6[-84.2,24.99] | 0.29 | 80.81[9.28,152.35] | 0.03 | 38.27[-30.5,107.04] | 0.27 |
| **Left Hemisphere- Stratified No PTE** | 1.53[-57.33,60.38] | 0.96 | -7.68[-74.38,59.03] | 0.82 | -5.15[-66.4,56.09] | 0.87 |
| **Right Hemisphere- Stratified No PTE** | 28.33[-20.74,77.39] | 0.26 | -19.37[-84.84,46.1] | 0.56 | -10.62[-74.97,53.72] | 0.75 |
